# Treatment pattern and health care resource utilization for Taiwanese patients with migraine: a population-based study

**DOI:** 10.3389/fneur.2023.1222912

**Published:** 2023-08-16

**Authors:** Yen-Feng Wang, Shuu-Jiun Wang, Yao Hsien Huang, Yung-Tai Chen, Yu-Chun Yen, Ben-Chang Shia, Ching-Wen Tsai, Hoi-Fong Chan, Tommaso Panni, Grazia Dell’Agnello

**Affiliations:** ^1^Department of Neurology, Neurological Institute, Taipei Veterans General Hospital, Taipei, Taiwan; ^2^College of Medicine, National Yang Ming Chiao Tung University, Taipei, Taiwan; ^3^Brain Research Center, National Yang Ming Chiao Tung University, Taipei, Taiwan; ^4^Eli Lilly and Company, Indianapolis, IN, United States; ^5^Department of Nephrology, Taipei City Hospital Heping Fuyou Branch, Taipei, Taiwan; ^6^Clinical Information Department, Quality Management Center, Taichung Veterans General Hospital, Taichung, Taiwan; ^7^Graduate Institute of Business Administration, College of Management, Fu Jen Catholic University, Taipei, Taiwan; ^8^Health Data Analytics and Statistics Center, Office of Data Science, Taipei Medical University, Taipei, Taiwan

**Keywords:** chronic migraine, episodic migraine, real-world, Taiwan, health care resource use, acute medication, preventive medication

## Abstract

**Objectives:**

Given the substantial disease burden, appropriate and effective management of migraine is a public health priority. To gain insights into real-world migraine management practices in Taiwan, current treatment patterns, costs, and health care resource use were assessed.

**Methods:**

This was a retrospective, longitudinal study using the Taiwan National Health Insurance Research Database. Included patients had an initial diagnosis of migraine (defined using International Classification of Diseases codes) between 1 January 2013 and 31 December 2017. Data analyzed included demographics; the use, number, and type of acute and preventive medications; and drug and medical services costs. Data were stratified according to migraine type (chronic [CM] or episodic [EM] migraine).

**Results:**

A total of 312,718 patients were included in the analyses: 53,992 (17.3%) had CM and 258,726 (82.7%) had EM. Most patients (81.7%) had used acute and/or preventive medications; acute medications used more frequently than preventive medications (78.0% vs. 20.2%). Acute medications were used by 81.6 and 77.3% of patients with CM and EM, respectively. Commonly used acute medications were acetaminophen (68.8%), ergots (49.4%), and non-steroidal anti-inflammatory drugs (38.4%); the use of triptans (6.0%), tramadol (3.1%), and other opioids (0.2%) was less common. A total of 28.6 and 18.5% of patients with CM and EM, respectively, used preventive medications. Flunarizine (68.9%), propranolol (40.7%), and topiramate (16.0%) were the most commonly used preventive medications. Most patients had used 1–2 acute or preventive medications, with the use of ≥3 acute or preventive medications more common in patients with CM than EM. Average total medical cost *per annum* was 4,169 New Taiwan Dollars (NTDs) per CM patient and 2,928 NTDs per EM patient, with CM patients having higher costs associated with medical service utilization and acute medication use.

**Conclusion:**

These real-world data suggest unmet needs for Taiwanese patients with migraine, including under-utilization of preventive medications and greater costs and health care resource use for patients with CM versus EM. These findings provide important information on treatment patterns, cost, and health care resource use for patients with migraine in Taiwan.

## 1. Introduction

Migraine is a neurological disease characterized by recurrent episodes of headache (1). The occurrence of ≤14 headache days a month is classified as episodic migraine (EM), whereas chronic migraine (CM) is characterized by ≥15 headache days a month, where at least 8 of those days meet the International Classification of Headache Disorders 3rd edition (ICHD-3) criteria for migraine (1). The global prevalence of migraine is 14.0% (2), and it is the leading cause of disability in persons under 50 years of age (3, 4). The reported prevalence in the Asia–Pacific region lies in the lower range of estimates for Caucasians (5). In Taiwan, the prevalence was estimated at 9.1% (6). Studies in Taiwan have reported associations between migraine and high levels of disability, comorbidity, lower quality of life, greater health care resource use, and loss of productivity (7–9). The consequence of migraine on the employed labor force in Taiwan is high absenteeism and, as a result, significant economic loss (10).

According to the current guidelines of the Taiwan Headache Society, triptans, acetaminophen, non-steroidal anti-inflammatory drugs (NSAIDs) and prochlorperazine injections are highly recommended medications in the acute treatment of migraine (11). However, it is recommended that treatment follows the concept of “stratified care”: for migraine attacks with moderate-to-severe disability (Migraine Disability Assessment [MIDAS] grade III or IV), triptans are recommended to be administered in the early stages of an attack, and ergotamine/caffeine compounds are a reasonable option. Oral NSAIDs can be used for patients with acute migraine attacks with none/little-to-mild disability (MIDAS grade I or II), and alternatives include combination analgesics, and intravenous/intramuscular NSAIDs (11). Currently, opioids, including tramadol, are not recommended for the routine treatment of acute migraine because of concerns about dependence, and lack of evidence supporting their use (11). Of the acute medications recommended in Taiwan, triptans and ergotamine are the only migraine-specific drugs (11). Other migraine-specific medications, gepants and ditans, may play a role in the treatment of migraine in Taiwan, but these agents have not yet been approved (11).

For preventive treatment of migraine, Taiwanese guidelines recommend propranolol, flunarizine, and topiramate as first-line therapies for EM, with valproic acid, amitriptyline, and anti-calcitonin gene-related peptide (CGRP) monoclonal antibodies suggested as second-line medications. Topiramate, flunarizine, onabotulinumtoxinA and anti-CGRP monoclonal antibodies are recommended as the first-line preventive medications for CM (12). When categorizing medications into first line or second line, considerations include efficacy, adherence, potential side effects, management of comorbidities, and cost (12).

Recently, several monoclonal antibodies targeting CGRP signaling have been developed with demonstrated efficacy for the prevention of migraine in both EM and CM, even after prior preventive treatments have failed and in patients with medication overuse (13–16). Two of these agents, galcanezumab and fremanezumab, have become available in Taiwan, although their coverage is limited to patients with difficult-to-treat CM according to the reimbursement regulations of Taiwan National Health Insurance. Thus, there remains an unmet need for Taiwanese patients with migraine (7).

Given the prevalence and the socioeconomic impacts of migraine, the appropriate and effective management of migraine represents an important public health priority. Therefore, a thorough understanding of migraine-related treatment patterns and health care resource utilization is important. The objective of our study was to assess current patterns of medication use, costs, and health care resource use in Taiwanese patients using data gathered from a national claims database, to gain an insight into real-world migraine management practices.

## 2. Methods

### 2.1. Study design

We performed a retrospective, longitudinal study using the Taiwan National Health Insurance Research Database, which is a claims-based dataset covering over 99% of the entire population. All patients included in the analysis had an initial diagnosis of migraine (defined as the index date) between 1 January 2013 and 31 December 2017. Migraine was defined using International Classification of Diseases (ICD) codes; initially ICD-9 codes were used, and then ICD-10 codes were used once Taiwan switched to ICD-10 use in 2016. CM was defined as an outpatient visit with 346.11 (ICD-9) or G43.7X (ICD-10); EM was defined as an outpatient visit with 346.x except 346.11 (ICD-9) or G43.X except G43.7X (ICD-10). The analyzed cohort could include patients who had an initial diagnosis of migraine and were later diagnosed with an additional headache disorder. Patients with a migraine diagnosis before 1 January 2013 were excluded. In addition, patients with a concomitant diagnosis of hypertension were excluded as it was difficult to ascertain whether beta-blockers were used for hypertension or migraine in these patients. The study period was defined as the period from the index date to 31 December 2017. The study protocol was approved by the Institutional Review Board of the National Yang Ming Chiao Tung University.

### 2.2. Medications

Acute medications included in the analysis were acetaminophen, ergots (including ergotamine, dihydroergotamine, and combinations with caffeine), NSAIDs, triptans (sumatriptan and rizatriptan), tramadol (including tramadol in combination with acetaminophen), and opioids other than tramadol (morphine, codeine, oxycodone, fentanyl, buprenorphine, and hydromorphone). Regarding tramadol and opioids other than tramadol, only tablet preparations were included, and prescriptions of other formulations, such as injections, tincture, and syrup, were excluded from the analysis. Preventive medications analyzed included the following classes: beta-blockers (propranolol, bisoprolol, atenolol, and metoprolol), anti-epileptic drugs (divalproex/valproate and topiramate), calcium channel blockers (flunarizine and verapamil), tricyclics (amitriptyline and imipramine), and others (venlafaxine, doxepin, clomipramine, candesartan, and gabapentin).

### 2.3. Data analyses

Demographic data included the year of diagnosis, region in Taiwan, age, hospital type (medical center, regional or district hospital, general clinic, or home care) and department (neurology or non-neurology) where the initial diagnosis of migraine was made. The use, number (1, 2, 3, or ≥ 4) and type of acute and preventive medications (drug class) were analyzed. Inclusion criterion for acute medication use was that it had been used at least once. Inclusion criterion for preventive medication use was that it had been used continuously for more than 28 days with no interval between prescriptions. Cost data included: total cost (defined as drug and medical costs [outpatient visits, emergency room visits, and admissions/inpatient services]); total cost per outpatient; emergency room or inpatient visit; number of days of acute medication use; and cost of acute medication by type. Costs were calculated based on the 365 days following the date of first diagnosis with migraine, and all data were expressed as per patient *per annum*. Data were grouped by patients with CM or EM and descriptive statistics performed using SAS version 9.4 for Windows.

## 3. Results

### 3.1. Patient cohort and demographics

**Figure 1:**
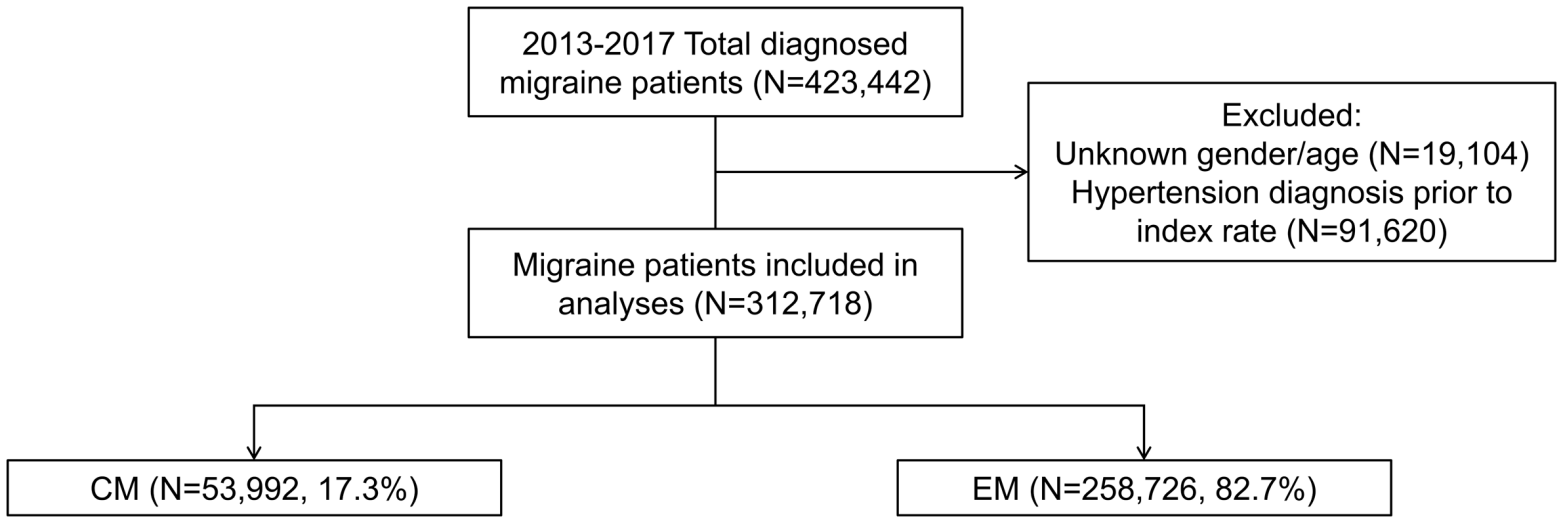
Patient cohort. Index date was defined as the date of first diagnosis with migraine between 1 January 2013 and 31 December 2017. CM was diagnosed according to ICD-9:346.11 or ICD-10:G43.7X. EM was diagnosed according to ICD-9:346.X except 346.11 or ICD-10:G43.X except G43.7X. CM, chronic migraine; EM, episodic migraine; N, number of patients.

Of the 423,442 patients who received an initial diagnosis of migraine (index date) between 1 January 2013 and 31 December 2017, 110,724 were excluded from the analysis due to missing data on gender or age or having had a hypertension diagnosis prior to the index date. Of the resulting 312,718 patients included in the analyses, 53,992 (17.3%) had CM and 258,726 (82.7%) had EM (Figure 1). The age group with the highest number of patients was 18–30 years, followed by 31–40 years, and 41–50 years. Primary care clinics were the most common clinical setting where patients had been diagnosed, followed by regional hospitals and medical centers. In 60.5% of patients, the diagnosis was initially made by health care providers or specialists other than neurologists (Table 1).

**Table 1:** Patient demographics.

Variable	CM		EM		All migraine	
	*N*	%	*N*	%	*N*	%
Number of patients, *N* (as a % of “All migraine”)	53,992	17.3	258,726	82.7	312,718	100
Region, *n* (as a % of group)						
North	22,976	42.6	115,323	44.6	138,299	44.2
Central	9,011	16.7	69,399	26.8	78,410	25.1
South	20,849	38.6	67,132	25.9	87,981	28.1
East	1,156	2.1	6,872	2.7	8,028	2.6
Age in years, *n* (as a % of group)						
<18	3,509	6.5	28,244	10.9	31,753	10.2
18–30	11,822	21.9	75,486	29.2	87,308	27.9
31–40	14,492	26.8	64,841	25.1	79,333	25.4
41–50	12,097	22.4	46,595	18.0	58,692	18.8
51–60	8,232	15.2	29,501	11.4	37,733	12.1
61–70	3,012	5.6	10,538	4.1	13,550	4.3
≥71	828	1.5	3,521	1.4	4,349	1.4
Hospital type, *n* (as a % of group)						
Medical center	7,286	13.5	51,234	19.8	58,520	18.7
Regional hospital	9,332	17.3	69,078	26.7	78,410	25.1
District hospital	5,486	10.2	35,780	13.8	41,266	13.2
Primary care clinic	31,693	58.7	100,366	38.8	132,059	42.2
Home care	0	0.0	8	0.0	8	0.0
Unknown	195	0.4	2,260	0.9	2,455	0.8
Department for first-time diagnosis, *n* (as a % of group)						
Neurology	19,474	36.1	104,130	40.2	123,604	39.5
Non-neurology	34,518	63.9	154,596	59.8	189,114	60.5

CM, chronic migraine; EM, episodic migraine; N, number of patients.

### 3.2. Acute and preventive medication use

**Figure 2:**
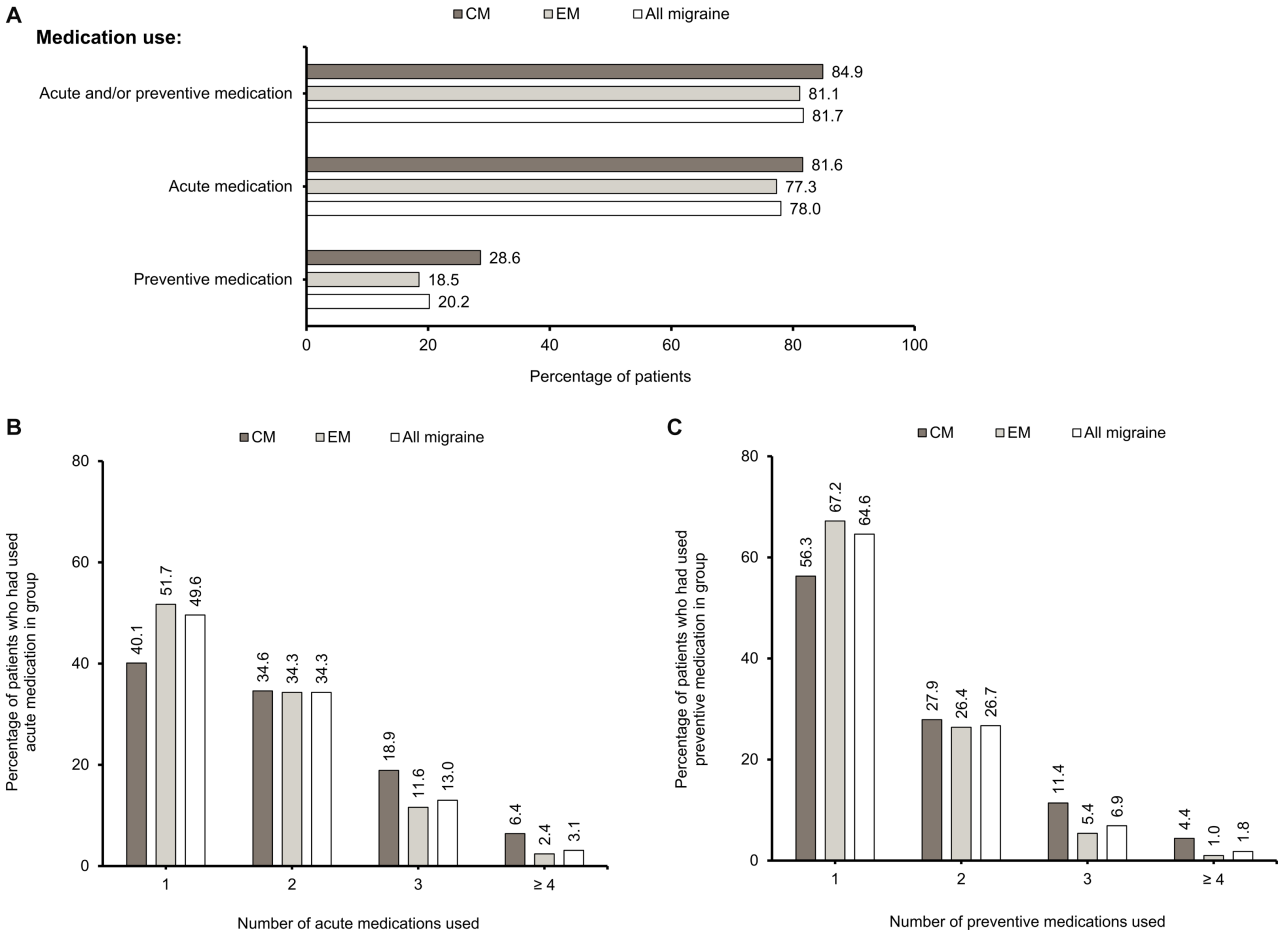
Acute and preventive medication use. **(A)** Percentages of CM, EM, and “All migraine” patients who had used acute and/or preventive, acute, and preventive medications. These categories are non-exclusive. Percentages of CM, EM, and “All migraine” patients who had never used acute and preventive medications are also shown. Inclusion criterion for acute medication use was that it had been used at least once. Inclusion criterion for preventive medication use was that it was used continuously for more than 28 days with no interval between prescriptions. **(B)** Percentages of CM, EM, and “All migraine” patients who had used 1, 2, 3, or ≥ 4 acute medications. **(C)** Percentages of CM, EM, and “All migraine” patients who had used 1, 2, 3, or ≥ 4 preventive medications. CM, chronic migraine; EM, episodic migraine; N, number of patients.

A high percentage of patients overall (81.7%) had used acute and/or preventive medications, with acute medications used more frequently than preventive medications (78.0% vs. 20.2%) (Figure 2A). Among the patients who had used acute medications, approximately half (49.6%) had used only one, 34.3% had used two, 13.0% had used three, and 3.1% had used four or more classes of acute medications (Figure 2B). Among patients who had used preventive medication, nearly two-thirds (64.6%) had used only one, 26.7% had used two, 6.9% had used three and 11.8% having used four or more classes of preventives (Figure 2C).

The proportions of patients with acute (81.6% vs. 77.3%) and preventive medication (28.6% vs. 18.5%) use were higher in CM than in EM (Figure 2A). A higher percentage of patients with CM had used ≥3 acute or preventive medications than those with EM. A higher percentage of CM patients had used ≥4 classes of medications compared with EM patients for acute (6.4% vs. 2.4%) and preventive (4.4% vs. 1.0%) treatment (Figures 2B,C). Overall, there is an increasing proportion of CM patients as the number of acute and preventive medications used increases (Supplementary Figure S1).

**Figure 3:**
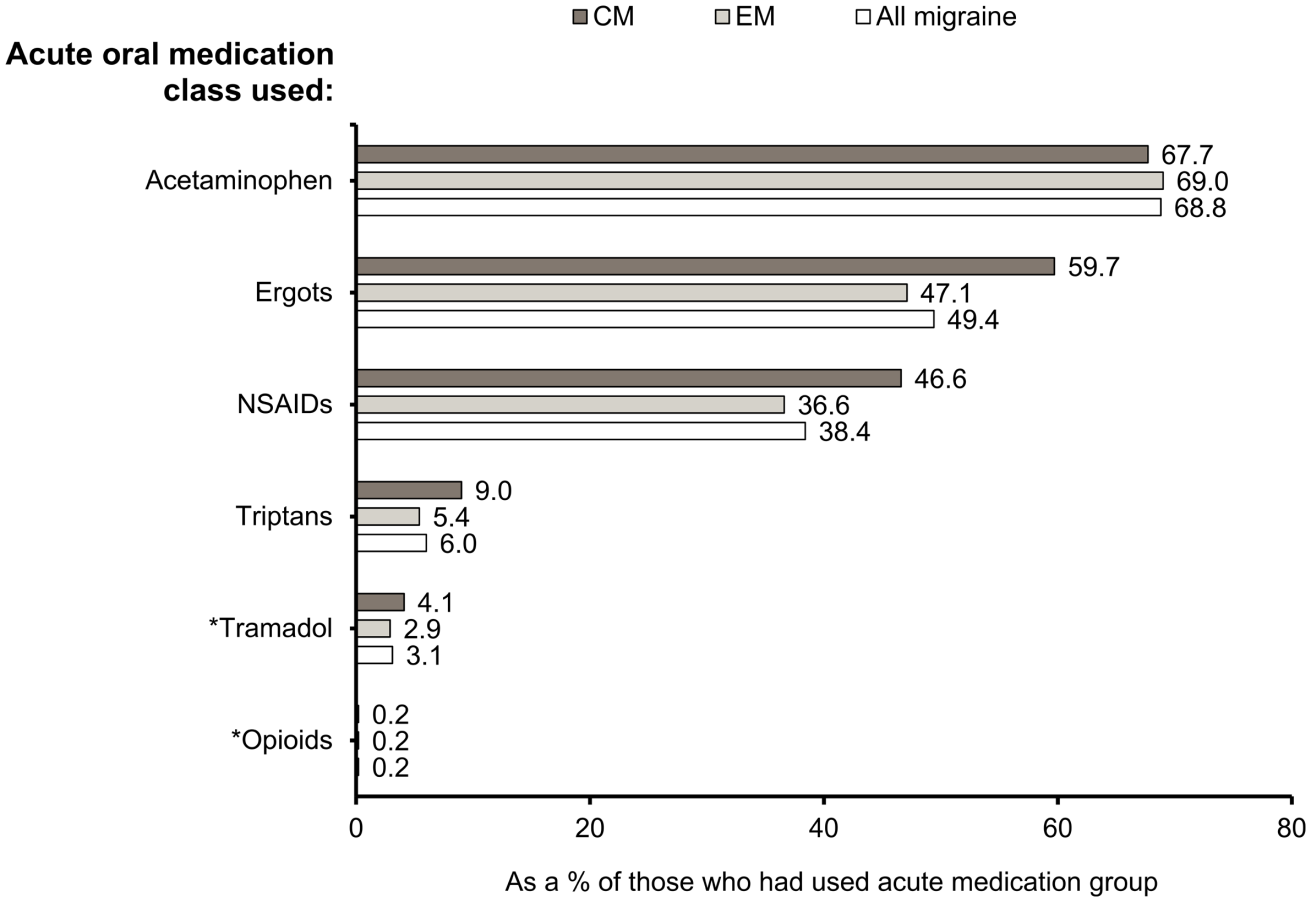
Drug classes of acute oral medications used. Percentages of CM, EM, and “All migraine” patients who had used specific acute medication classes. *Opioids were further grouped as “tramadol” and “opioids other than tramadol.” CM, chronic migraine; EM, episodic migraine; N, number of patients; NSAID, non-steroidal anti-inflammatory drug.

Acetaminophen was the most frequently used acute medication overall (68.8%), followed by ergots (49.4%), and NSAIDs (38.4%). Overall, 6.0% of patients had used triptans. Fewer patients had used tramadol (3.1%) and opioids other than tramadol (0.2%). A higher proportion of patients with CM than EM had used ergots (59.7% vs. 47.1%), NSAIDs (46.6% vs. 36.6%), and triptans (9.0% vs. 5.4%), whereas the percentages of patients prescribed with acetaminophen were similar (67.7% vs. 69.0%) (Figure 3). When each of the acute medication classes was analyzed separately, triptan users had the highest proportion of CM patients (26.8%) (Supplementary Figure S1).

**Figure 4:**
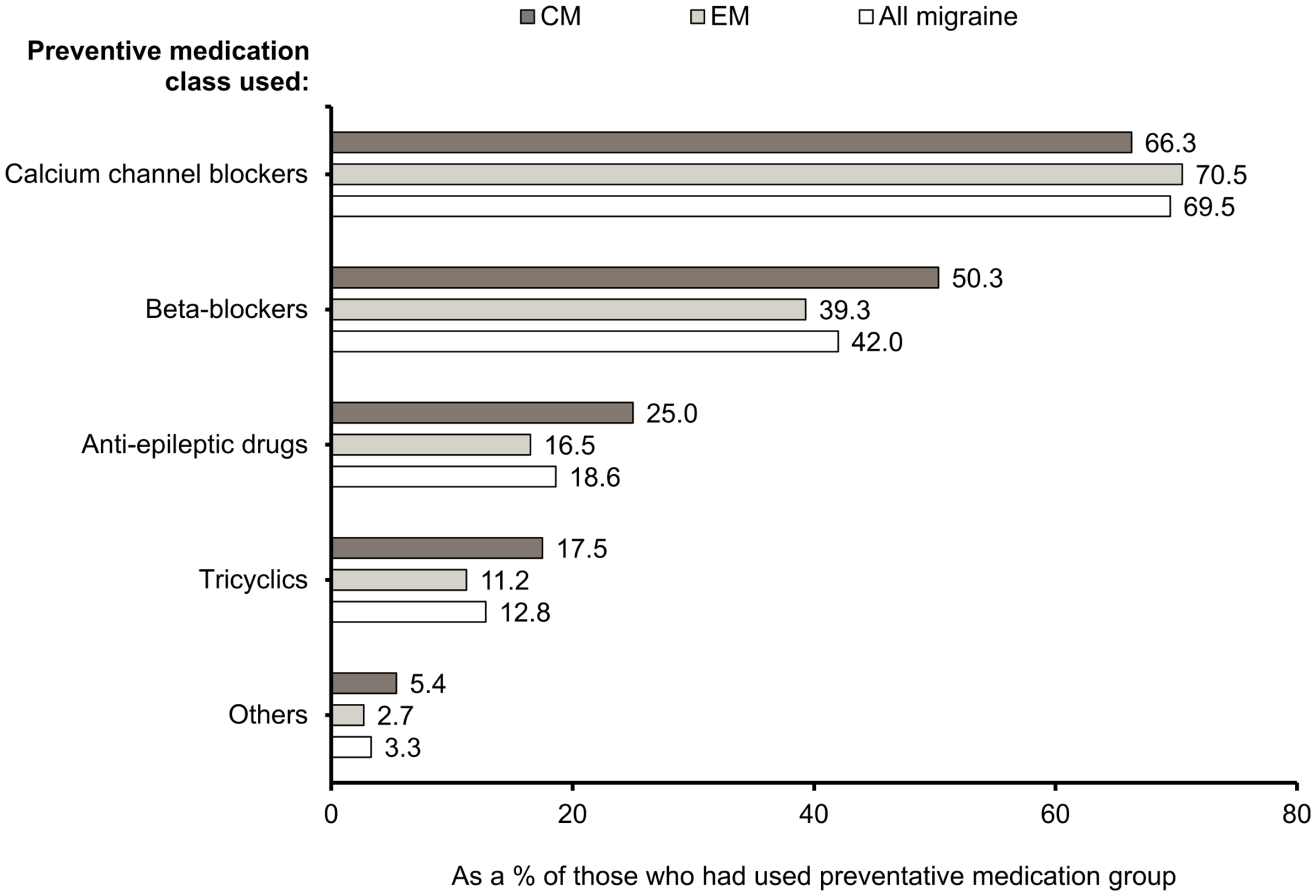
Classes of preventive medications used. Percentages of CM, EM, and “All migraine” patients who had used specific preventive medication classes (see Supplementary Table S1 for further information). CM, chronic migraine; EM, episodic migraine; N, number of patients.

Calcium channel blockers were the most commonly used preventive medication class in patients overall (69.5%) followed by beta-blockers (42.0%), anti-epileptic drugs (18.6%), and tricyclics (12.8%) (Figure 4). When data for individual drugs were analyzed, flunarizine (68.9%), propranolol (40.7%), and topiramate (16.0%) were the most commonly used preventive medications in Taiwan (Supplementary Table S1). The percentages of patients who had used each of the preventives were higher in CM than in EM, except for calcium channel blockers, which were used by similar proportions of patients with CM and EM (Figure 4).

### 3.3. Cost and health care resource use

**Figure 5:**
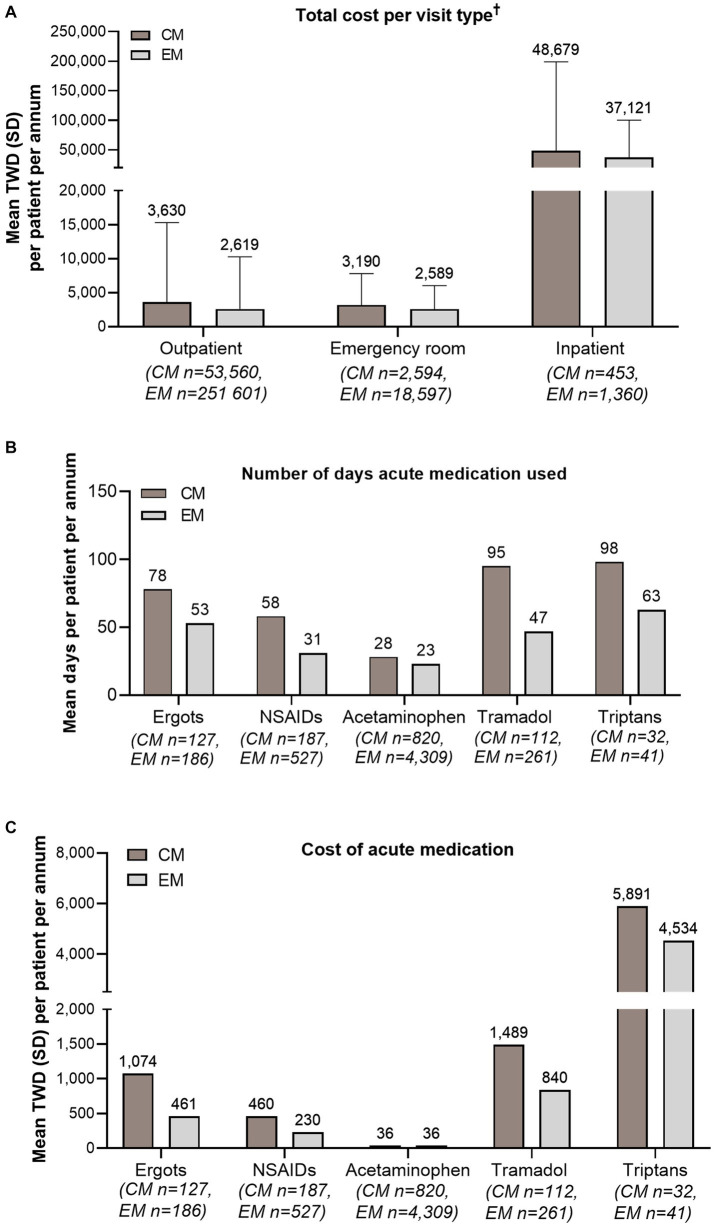
Cost and health care resource use. **(A)** Mean total cost (TWD) per CM and EM patient *per annum*, per visit type. **(B)** Mean number of days on which acute medication was used per CM and EM patient *per annum*. **(C)** Mean cost (TWD) of acute medication per CM and EM patient *per annum*. ^†^Includes both drug and non-drug costs (outpatient visits, emergency room visits, and admissions/inpatient services). Costs were calculated based on the 365 days following the date of first diagnosis with migraine. Error bars represent SD. CM, chronic migraine; EM, episodic migraine; N, number of patients; NSAID, non-steroidal anti-inflammatory drug; SD, standard deviation; TWD, Taiwan Dollar.

The average total medical cost *per annum* was New Taiwan Dollar (NTD) 4,169 per CM patient and NTD 2,928 per EM patient. Among those who had outpatient, emergency, or inpatient visits, the cost per patient *per annum* for all visits was higher for CM than EM (Figure 5A). Among patients who had used ergots, NSAIDs, tramadol, and triptans for acute treatment, CM patients had more days per year prescribed with each of these medications and therefore greater costs, compared with EM patients (Figures 5B,C). For acetaminophen, its use was somewhat higher in CM patients than EM patients, but the costs were similar between CM and EM.

## 4. Discussion

This analysis of the Taiwanese National Claims Database, which covers 99% of the population, was able to define a profile of health care resource utilization of migraine patients in Taiwan. Migraine tended to affect those aged 18–30 years the most (27.9%), followed by those aged 31–40 (25.4%), 41–50 (18.8%), and 51–60 (12.1%) years, and patients with EM (82.7%) constituted a higher proportion than those with CM (17.3%). Overall, the initial diagnosis was made by non-neurologists in most patients (60.5%), with a primary care clinic being the most common practice setting. More than 80% of patients had used either acute or preventive medications for migraine. However, preventive medications were prescribed in only 20.2% of migraine patients and, even in CM patients, the percentage was only 28.6%. Patients with CM used more medications for migraine than patients with EM and consequently their migraine-related costs, both for medical visits and medications, were higher. It is noteworthy that the use of opioids other than tramadol was very rare (0.2%) in Taiwanese migraine patients.

Given that only one-fifth of all patients had used preventive medication in this analysis, this suggests an under-utilization of these medications. Under-treatment of migraine is a common issue. The US-based CaMEO study reported a similar finding, with 80.2% (5,275) of the 6,579 respondents who reported ≥4 monthly headache days having never used a daily oral migraine preventive (17). A German database study reported that 29.1% of patients received at least one prescription for preventive medication (18), while in the Eurolight study 1.6–13.7% of patients received preventives (19). In the OVERCOME study, while 40.4% of US participants were eligible for migraine preventive medication, only 16.8% were current users (20), and data from Japan showed that use of preventive medications was even lower, at 9.2% (21). In the present study, the percentage of patients receiving preventive treatment was as low as 20.2%, and it was only 28.6% even in CM patients. In Taiwan, a referral mechanism is included in the Taiwan National Health Insurance; however, it is not mandatory. In addition to being referred by primary care physicians or other specialties, patients can also make appointments with neurologists or headache specialists directly, and copayment associated with direct consultations without a referral is typically minimal. In addition, all oral preventive medications are covered by the health system in Taiwan, and there is no restriction on their use. These findings indicate the presence of an unmet need for migraine patients that is probably not attributable to limited access to medical resources. More effort is needed to increase the awareness of migraine and its treatment in the general public and among medical professionals.

Flunarizine is the predominant preventive medication prescribed for migraine patients in Taiwan, and it was used by approximately two-thirds of EM or CM patients in the current study. Interestingly, although flunarizine was not recommended as a first-line agent for either EM or CM patients in the 2017 Taiwanese guidelines (22), its use was still widespread. Flunarizine was listed among the first-line choices for migraine prophylaxis in the European guidelines at that time (23), and studies show that it may be as effective as other preventives (24–30). In addition, it is easy to use; in contrast with beta blockers, there is no requirement for heart rate and blood pressure monitoring (31), and unlike topiramate, there is no need for gradual titration (32).

Management of patients who have failed multiple preventives is challenging in clinical practice. In the present study, a substantial minority of CM (4.4%) and EM (1.0%) patients had used ≥4 classes of preventives (Figures 2B,C). It is possible that the change in preventive treatments could be attributed to lack of efficacy or intolerable side effects. Therefore, the identified population might correspond to what is called “difficult-to-treat” migraine patients in the literature (33, 34), and these individuals could be potential candidates for onabotulinumtoxinA or CGRP monoclonal antibodies according to the reimbursement regulations of the Taiwan National Health Insurance. However, it is difficult to ascertain the reasons for the change in preventive treatment in the claims database, and further studies are needed to confirm these estimates.

Triptan use appeared to be low in Taiwan, and the percentage of triptan users (6.0%) in the present study was in the lower range of estimates reported in prior studies (3.4–22.7%) (18–21). Although triptans are listed among the first-line choices in the Taiwanese guidelines, acetaminophen, ergots, and NSAIDs were much more commonly used in clinical practice. Similar to the present study, the use of simple analgesics, NSAIDs, and over-the-counter medications was also common in reports from other countries (18, 19, 21). The finding that triptan use was limited to a small proportion of patients in Taiwan could be attributed mainly to the cost of these medications, and that only two triptans are available in our country, namely sumatriptan and rizatriptan. Sumatriptan is much more commonly used than rizatriptan and is available only in its brand-name forms as oral tablets and nasal sprays. In contrast, only one generic preparation of rizatriptan oral tablets is available. The difference in cost between triptans and other acute medications is considerable. A 50 mg tablet of sumatriptan typically costs $5 US dollar (USD) versus $1.8 USD for a 5 mg generic rizatriptan tablet. However, it costs only $0.05 USD for an ergotamine-caffeine combination tablet. Therefore, medical costs associated with triptan use were much higher than those for other acute medications. Also, since triptan use was limited, some physicians or patients might not be familiar with these agents, or might even have excessive concerns about potential adverse effects, i.e., triptanophobia (35). These reasons could also have hindered the widespread use of triptans.

Of note, the use of opioids other than tramadol was very rare in Taiwan (<0.5%), and the use of tramadol was seen in <5% of patients. This proportion is low compared with a study from Germany, which reported that opioids were one of the most commonly prescribed acute medications (19.1% of acute medication prescriptions), although opioids were generally not recommended in German guidelines (18). A survey of migraine patients in the US found that 32.5% of respondents with acute prescription medication for headache/migraine in the previous 3 months reported the use of opioids (36). Besides, in the American Migraine Prevalence and Prevention (AMPP) study, nearly 30% people with migraine included in the study were either previous users of opioids, or were current opioid users. Among current users, 16.6% met the criteria for opioid dependence (37). In the AMPP study, opioid use was associated with more severe headache-related disability, symptomology, and comorbidities (37). Furthermore, the increased use of prescription opioids has been found, among other factors, to be associated with male gender, chronic migraine, more severe disability, and anxiety and depression (36). As well as furthering levels of disability and decreasing quality of life (QoL), chronic opioid therapy has been reported to correlate with psychiatric comorbidities (38). Although opioids can be effective to treat migraine, they can also exacerbate migraine and potentially lead to CM (39). Additionally, the potential for abuse of opioids is great, with a high risk of addiction when misused or taken for long periods (40). Opioids should therefore be used sparingly, and only when in conjunction with comprehensive assessment and the integration of psychological treatment (38). In Taiwan, opioids are not recommended in the guidelines (11, 12) because of potential side effects and the risk of overuse and addiction, which could have contributed to the extremely low proportions of opioid use in Taiwanese patients with migraine. In fact, this might not be a unique phenomenon. The prescription rates of opioids for various indications are also relatively low in Asian countries, which could be attributed to cultural differences, concerns about opioid use in the general public and medication professionals, and tight regulations on their use (41).

Migraine, particularly CM, is associated with a substantial burden, both for the patient and for society. It was shown that migraine in Taiwan was associated with high levels of migraine-related disability and great impacts on health-related QoL, as well as substantial productivity losses with respect to millions of lost workdays and high health care utilization costs (7, 10), and the disease burden was greater in CM compared with EM (7). The findings in the present study were in keeping with our prior report (7). However, the present study not only extended the findings to a population level, but also provided updated estimates for medical costs associated with health care resource utilization. The findings highlight the importance of increasing migraine awareness, as well as optimization of the treatment of migraine patients, especially those with CM.

### 4.1. Strengths and limitations

One of the most important strengths of this study is the use of a nationwide database. The analysis consisted of a large sample size, which could potentially minimize selection bias. Also, compared with clinical trials, the results were derived from an unselected migraine population without a predefined treatment protocol, and therefore the findings are likely to reflect real-world practice more accurately. Limitations of this study are that although all patients included in the analysis had an initial diagnosis of migraine, the cohort could include some patients who had an initial diagnosis of migraine and were later diagnosed with an additional headache disorder; therefore, we cannot be certain that medication use, cost, and health care utilization all attributed to the initial migraine diagnosis. In particular, some of the medications, such as acetaminophen and NSAIDs, are non-prescription drugs, and over-the-counter preparations are available from pharmacies. Therefore, the costs related to medication use could have been underestimated. In addition, the diagnosis of CM was not formally included in the ICD-9 coding system, and the actual headache frequency was not available in the claims database. Further detailed analysis was precluded by the retrospective nature of the study. Therefore, coding inaccuracy and misclassification bias could be important concerns. However, the percentage of CM among migraine patients was close to estimates from studies in primary care or based on claims database (42, 43). Of note, only the total costs and costs associated with medications were available in the dataset used for the current analysis and further details, such as the cost of instrumental examinations or specialist visits, could not be analyzed. Finally, since the end date of the current study was 2017, the analysis also did not include more recent treatment options, such as onabotulinumtoxinA and CGRP-targeted agents, which were not available until 2020 and 2021, respectively. Since these agents are of proven efficacy in clinical trials and real-world studies, how they could change patterns of treatment and health care utilization or even narrow the gap of unmet needs deserves to be further studied in the future.

## 5. Conclusion

These data from the Taiwan National Health Insurance Research Database suggest unmet needs for Taiwanese patients with migraine, including an under-utilization of preventive medications. In addition, there were greater costs and health care resource use for patients with CM compared with EM. These findings provide important information on treatment patterns, cost, and health care resource use for patients with migraine, which may help to achieve appropriate and effective management of migraine for Taiwanese patients. Future studies will be able to investigate whether the availability of monoclonal antibodies targeting CGRP has changed the treatment landscape for patients with migraine in Taiwan.

## Data availability statement

The data analyzed in this study is subject to the following licenses/restrictions: The data analyzed in this study was obtained from the National Health Insurance Research Database, which has been transferred to the Health and Welfare Data Science Center (HWDC). Interested researchers must obtain the data through formal application to the HWDC, Department of Statistics, Ministry of Health and Welfare, Taiwan. Requests to access these datasets should be directed to https://dep.mohw.gov.tw/DOS/cp-5119-59201-113.html.

## Ethics statement

The studies involving humans were approved by the National Yang-Ming University. The studies were conducted in accordance with the local legislation and institutional requirements. Written informed consent for participation was not required from the participants or the participants’ legal guardians/next of kin because The Institutional Review Board (IRB) approved research protocol is mandatory to use and analyze the National Health Insurance Research Database (NHIRD) data. All patient data captured in NHIRD are delinked and deidentified. The use and analysis of NHIRD does not require investigators to obtain written informed consent nor a waiver. The National Yang Ming University IRB committee approved the protocol before proceeding with the study.

## Author contributions

Y-FW and B-CS were involved in the conception and design of the work, acquisition, analysis, and interpretation of the data for the work. S-JW was involved with the conception of the work and the acquisition of the data for the work. YH and H-FC were involved with the interpretation of the data for the work. Y-TC was involved with the conception of the work and analysis of the data for the work. Y-CY was involved with the acquisition and analysis of the data for the work. C-WT was involved with the analysis of the data for the work. TP was involved with the analysis and interpretation of the data for the work. GD was involved with the design of the work and the interpretation of the data for the work. All authors provided critical revision of the manuscript for important intellectual content and have participated sufficiently in the work to agree to be accountable for all aspects of the work in ensuring that questions related to the accuracy or integrity of any part of the work are appropriately investigated and resolved and give their final approval of the manuscript to be published.

## Funding

This research was funded by Eli Lilly and Company.

## Conflict of interest

H-FC, TP, YH, and GD are full-time employees of Eli Lilly and Co. Y-FW has received honoraria as a speaker from Taiwan branches of Allergan/AbbVie, Eli Lilly, Novartis, Pfizer, Sanofi, UCB, Viatris, Orient EuroPharma, Chugai, and Teva. He has received research grants from the Taiwan Ministry of Science and Technology, and Taipei Veterans General Hospital. S-JW has served on the advisory boards of Daiichi-Sankyo, Eli Lilly and Novartis; has received honoraria as a moderator from Allergan/AbbVie, Pfizer, Eli Lilly, Biogen and Eisai and has been the PI in trials sponsored by Eli Lilly, Novartis, and Allergan/AbbVie. He has received research grants from the Taiwan Minister of Technology and Science (MOST), Brain Research Center, National Yang Ming Chiao Tung University from The Featured Areas Research Center Program within the framework of the Higher Education Sprout Project by the Ministry of Education (MOE) in Taiwan, Taipei Veterans General Hospital, Taiwan Headache Society and Taiwan branches of Eli Lilly, Novartis, and Pfizer.

The remaining authors declare that the research was conducted in the absence of any commercial or financial relationships that could be construed as a potential conflict of interest.

## Publisher’s note

All claims expressed in this article are solely those of the authors and do not necessarily represent those of their affiliated organizations, or those of the publisher, the editors and the reviewers. Any product that may be evaluated in this article, or claim that may be made by its manufacturer, is not guaranteed or endorsed by the publisher.
