# Supplementary material for: Treatment pattern and health care resource utilization for Taiwanese patients with migraine: a population-based study
Source: Front Neurol. 2023 Aug 16;14:1222912. doi: 10.3389/fneur.2023.1222912 (PMC10466390; doi:10.3389/fneur.2023.1222912)
Supplement: Supplementary file 1 [file Table_1.DOCX]

**Supplementary Table 1.** Drugs within classes of preventive medications used. Percentages of CM, EM, and “All migraine” patients who had used specific preventive medication classes and drugs.

|  | **CM** |  | **EM** |  | **All migraine** |  |
| --- | --- | --- | --- | --- | --- | --- |
| **Variable** | **N** | **%** | **N** | **%** | **N** | **%** |
| Number of patients, N (as a % of “All migraine”) | 53,992 | 17.3 | 258,726 | 82.7 | 312,718 | 100 |
| Preventive medication use, n (as a % of group) | 15,426 | 28.6 | 47,834 | 18.5 | 63,260 | 20.2 |
| Preventive medication type, n (as a % of those using preventive medication) |  |  |  |  |  |  |
| Calcium channel blockers^a^ | 10,230 | 66.3 | 33,708 | 70.5 | 43,938 | 69.5 |
| Flunarizine | 10,167 | 65.9 | 33,431 | 69.9 | 43,598 | 68.9 |
| Verapamil | 172 | 1.1 | 432 | 0.9 | 604 | 1.0 |
| Beta-blockers^a^ | 7,761 | 50.3 | 18,822 | 39.3 | 26,583 | 42.0 |
| Propranolol | 7,559 | 49.0 | 18,194 | 38.0 | 25,753 | 40.7 |
| Bisoprolol | 201 | 1.3 | 504 | 1.1 | 705 | 1.1 |
| Atenolol | 111 | 0.7 | 306 | 0.6 | 417 | 0.7 |
| Metoprolol | 99 | 0.6 | 89 | 0.2 | 188 | 0.3 |
| Anti-epileptic drugs^a^ | 3,860 | 25.0 | 7,875 | 16.5 | 11,735 | 18.6 |
| Topiramate | 3,316 | 21.5 | 6,788 | 14.2 | 10,104 | 16.0 |
| Divalproex/valproate | 1,059 | 6.9 | 1,633 | 3.4 | 2,692 | 4.3 |
| Tricyclics^a^ | 2,706 | 17.5 | 5,377 | 11.2 | 8,083 | 12.8 |
| Amitriptyline | 1,208 | 7.8 | 2,038 | 4.3 | 3,246 | 5.1 |
| Imipramine | 1,498 | 9.7 | 3,339 | 7.0 | 4,837 | 7.6 |
| Others^a^ | 828 | 5.4 | 1,285 | 2.7 | 2,113 | 3.3 |
| Venlafaxine | 662 | 4.3 | 774 | 1.6 | 1,436 | 2.3 |
| Doxepin | 139 | 0.9 | 242 | 0.5 | 381 | 0.6 |
| Clomipramine | 9 | 0.1 | 34 | 0.1 | 43 | 0.1 |
| Candesartan | 27 | 0.2 | 45 | 0.1 | 72 | 0.1 |
| Gabapentin | 125 | 0.8 | 338 | 0.7 | 463 | 0.7 |

^a^Patients may have received more than one treatment from this treatment class.

CM: chronic migraine; EM: episodic migraine; N: number of patients.
