# Supplementary material for: Treatment pattern and health care resource utilization for Taiwanese patients with migraine: a population-based study
Source: Front Neurol. 2023 Aug 16;14:1222912. doi: 10.3389/fneur.2023.1222912 (PMC10466390; doi:10.3389/fneur.2023.1222912)

**Supplementary Figure 1.** Percentages of patients with CM and EM among users of acute and preventive medications. Proportions of CM/EM patients stratified by the number of acute (A) and preventive (B) medication used, and stratified by the category of acute (C) and preventive (D) medications used. The tricyclics category includes amitriptyline and imipramine, and the Other category includes candesartan and venlafaxine. CCBs, calcium channel blockers; CM, chronic migraine; EM, episodic migraine; NSAIDs, non-steroidal anti-inflammatory drugs.


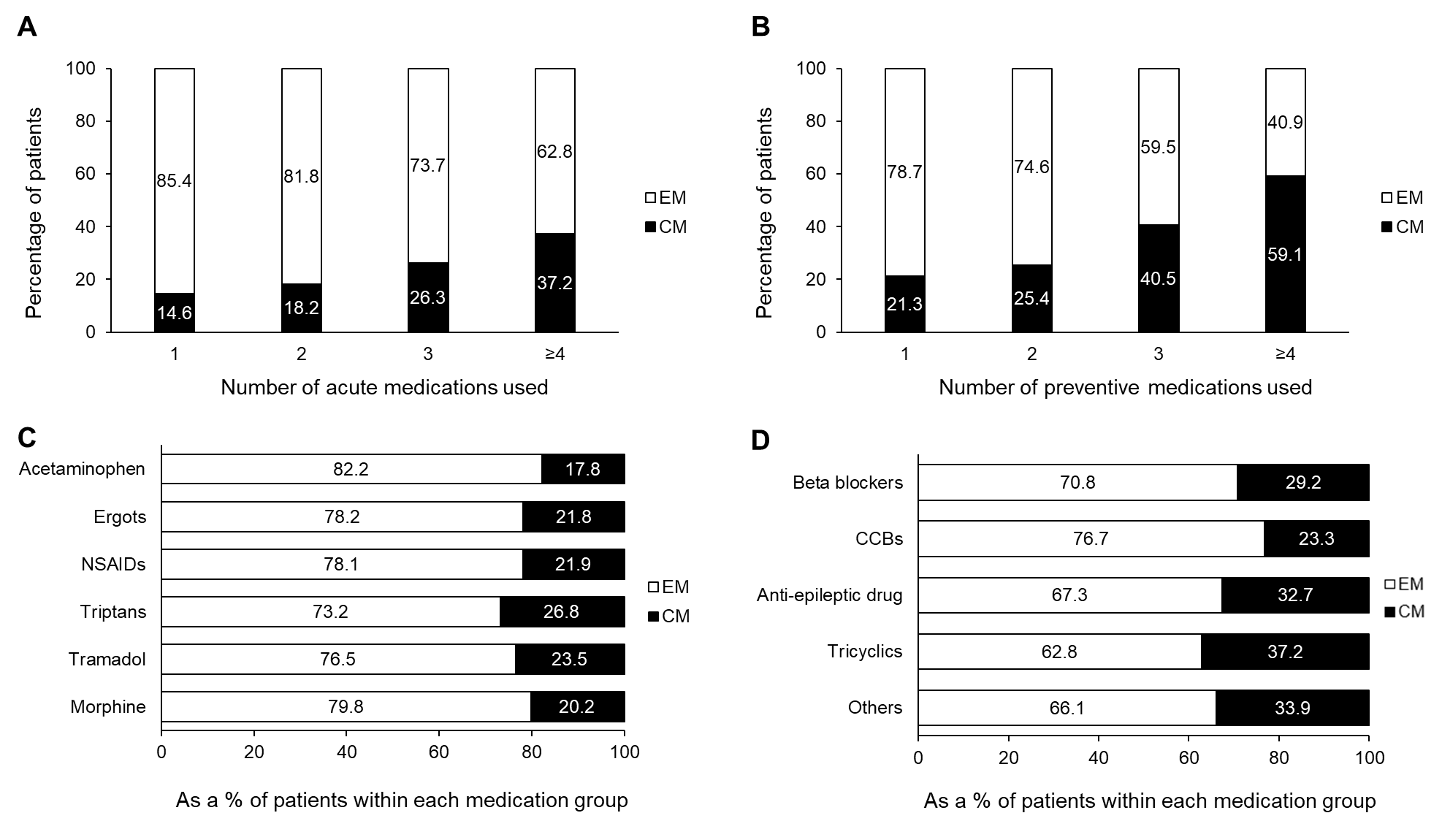

Supplement: Supplementary file 2 [file Data_Sheet_1.DOCX]
